# New insights on effects of a dietary supplement on oxidative and nitrosative stress in humans

**DOI:** 10.1002/fsn3.178

**Published:** 2014-10-17

**Authors:** Boris V Nemzer, Nelli Fink, Bruno Fink

**Affiliations:** 1 VDF FutureCeuticals Inc.2692 N State Rt. 1-17, Momence, Illinois, 60954; 2 University of Illinois at Urbana-Champaign1201 W. Gregory Dr, Urbana, Illinois, 61801; 3 Noxygen Science Transfer & Diagnostics GmbHLindenmatte 42, 79215, Elzach, Germany

**Keywords:** Dietary supplement, EPR, inflammatory response, nitric oxide, oxidative stress, RONS, SPECTRA™, vitality test

## Abstract

The research community is generally agreed that maintenance of healthy levels of free radicals and related oxidants are important for good health. However, utilization of the “redox stress hypothesis” can provide us with concrete nutritional targets in order to better support and maintain “optimal health.” Following this hypothesis we performed a crossover, double-blind, placebo-controlled, single-dose study on the effects of SPECTRA™, a dietary supplement, on oxidative stress markers (OSM) in human participants (*n* = 22). The measurement of OSM (ex vivo intra- and extracellular formation of reactive oxygen species (ROS, O_2_^−^, H_2_O_2_, OH^−^) in whole blood, respiratory activity of blood cells, as well as mitochondrial-dependent ROS formation, and respiratory activity), was performed using EPR spectrometer *nOxyscan*, spin probe CMH, and oxygen label NOX-15.1, respectively. Furthermore, we investigated the ability of SPECTRA™ to modulate ex vivo cellular inflammatory responses induced by stimulation with exogenous TNF-*α* and also followed changes in bioavailable NO concentrations. In this clinical study, we demonstrated that administration of SPECTRA™ resulted in statistically significant long-term inhibition of mitochondrial and cellular ROS generation by as much as 17% as well as 3.5-times inhibition in extracellular NADPH system-dependent generation of O_2_^−^, and nearly complete inhibition of extracellular H_2_O_2_ formation. This was reflected in more than two times inhibition of ex vivo cellular inflammatory response and also increases in bioavailable NO concentration. For the first time, we have measured synergetic, biological effects of a natural supplement on changes in OSM and cellular metabolic activity. The unique design and activity of the plant-based natural supplement, in combination with the newly developed and extended Vitality test, demonstrates the potential of using dietary supplements to modulate OSM and also opens the door to future research into the use of natural supplements for supporting optimal health.

## Introduction

During the last four decades, the research community has generally agreed that a dynamic, appropriately reactive, and healthy balance between levels of free radicals and levels of related oxidants is important for “optimal health.” Imbalances of free radicals, and potentially unhealthy levels of oxidants versus antioxidants, are collectively defined by the scientific community as “oxidative and nitrosative stress.” The delicate balance between beneficial and harmful effects caused by reactive oxygen and nitrogen species (RONS) is an important aspect of living organisms. Emerging research suggests that this balance may be achieved by a mechanism called “redox regulation.” This theory contends that the process of redox regulation protects living organisms from various oxidative stresses and maintains “redox homeostasis” by controlling the redox status in vivo (Dröge 2002). An exciting discovery (Sohal and Orr 2012) has refocused and refined this theory into the “redox stress hypothesis” of aging. In this new view, aging is the result of functional losses that are primarily caused by a progressive pro-oxidizing shift in the redox status of cells and tissues. This in turn leads to the overoxidation of redox-sensitive protein thiols and the consequent disruption of normal cellular functions. The “redox stress hypothesis” is based upon the status of the redox buffers of cells, tissue, and organisms. According to this theory, many of the components of our redox buffers are fundamental species of our antioxidant network. Just as it is essential to maintain our pH buffers, we must also maintain a healthy, and appropriately reduced oxidative state for our redox buffers. The effect of reactive oxygen species (ROS) that may cause potential biological damage has been termed “oxidative stress” and the effect of reactive nitrogen species (RNS) has been termed “nitrosative stress” (Kovacic and Jacintho 2001; Valko et al. 2001; Ridnour et al. 2005). This model presents us with concrete targets for potential nutritional intervention in order to maintain optimal health and support healthy aging and provides a means to investigate the direct effects of nutritional materials on biomarkers of significance for healthy aging (Broedbaek et al. 2013). The healthy balance in the body is comprised of four components:

1. An appropriately modulated, healthy flux of free radicals and oxidants.
2. An appropriate level of antioxidants coupled with fully functional systems to recycle these antioxidants.
3. Robust nutritional support that helps to maintain optimal levels of supportive antioxidants and cofactors.
4. Fully functioning enzyme systems that repair or recycle and replace damaged cellular materials, for example, DNA, RNA, enzymes, proteins, and endogenous redox molecules (glutathione, vitamin C, vitamin E etc.).

Electron paramagnetic resonance (EPR) spectroscopy is a technique that is recognized in the scientific community as a gold standard methodology (Dikalov et al. 2007) for direct observation ex vivo or in vivo of the formation of RONS. Recently, we also published observation of imaging of ROS (Ji et al. 2012) performed in living animals. It has been shown that for studies of intact tissues and cells, the cyclic hydroxylamine spin probes offer a distinct advantage over nitrone spin traps to measure the production of superoxide anion and other radicals due to the fact that they yield very stable products and strong EPR signals. Cyclic hydroxylamine spin probes such as 1-hydroxy-3-carboxy-pyrrolidine and 1-hydroxy-3-methoxycarbonyl-2.2.5.5-tetramethylpyrrolidine are very effective scavengers of superoxide radicals (Dikalov et al. 1998; Fink et al. 2000; Dikalov and Fink 2005). The major weaknesses of EPR are: (1) it is expensive and (2) it requires a substantial amount of space. To overcome these weaknesses, Noxygen Science Transfer & Diagnostics GmbH (Elzach, Germany) designed a bench-top EPR spectrometer “*nOxyscan*.” These advances in instrumentation provided us with an opportunity to perform a pilot study to investigate the bioactivity of a nutritional supplement SPECTRA™, a formulation consisting of high antioxidant activity fruit, vegetable concentrates, and herbal extracts, manufactured by FutureCeuticals, Inc. (Momence, IL) and standardized to a minimum of total antioxidant capacity (TAC) as measured by a series of oxygen radical absorbance capacity (ORAC)—based assays collected under the name ORAC 5.0, including ORAC, HORAC, NORAC, SORAC, and SOAC (Mullen et al. 2011).

## Material and Methods

### Natural SPECTRA™ total ORAC 5.0 blend

This full-spectrum antioxidant activity product is a proprietary combination of fruit, vegetable, and herb extracts and concentrates: broccoli powder and broccoli sprouts concentrate, onion extract, tomato concentrate, dried carrot, spinach, kale concentrate, brussel sprout concentrate, whole coffee fruits extract, acerola extract, camu camu powder, acai berry concentrate, mangosteen concentrate, green tea extract, apple extract, turmeric concentrate, garlic, basil concentrate, oregano, cinnamon concentrate, elderberry concentrate, blackcurrant extract, blueberry extract, sweet cherry powder, blackberry powder, chokeberry, raspberry powder, and bilberry extract. The ORAC 5.0 assay measures antioxidant activities against hydroxyl, peroxyl, peroxynitrite, singlet oxygen, and superoxide anion. SPECTRA™ is standardized to minimum 40,000 μmol trolox equivalent (TE) per gram of ORAC 5.0 assay (Nemzer et al. 2014).

### Sample preparation for antioxidant measurements

The sample preparation was conducted following the previous protocol (Mullen et al. 2011; Nemzer et al. 2014). Approximately 20 mg of SPECTRA™ was extracted with 20 mL of ethanol/water (70:30 v/v) for 1 h at room temperature on an orbital shaker. After centrifugation at 4164*g*, the supernatant of the extract was subjected to the TAC assay. The TAC includes the determination of radical scavenging capacities against five free radicals, namely, peroxyl, hydroxyl, peroxynitrite, superoxide anions, and singlet oxygen radicals. All results were expressed as Trolox equivalent per gram (*μ*mol TE/g) and the TAC was the sum of the five individual results.

### Peroxyl radical scavenging capacity (ORAC assay)

The ORAC assay was measured according to a previous report by Ou et al. (2002) and Huang et al. (2002) with modification. The FL600 microplate fluorescence reader (Bio-Tek Instruments, Inc., Winooski, VT) was used with an excitation wavelength of 485 (20 nm) and emission wavelength of 530 (25 nm). About 2,20-Azobis(2-amidinopropane) dihydrochloride (AAPH) was used to generate peroxyl radical. Fluorescein (FL) was used as a fluorescent probe to indicate the extent of damage from its reaction with the peroxyl radical. The antioxidant effect was measured by comparing the fluorescence time/intensity area under the curve of the sample to that of a control with no antioxidant. Trolox was prepared as the standard solution. Fluorescence was measured every min for up to 35 min.

### Hydroxyl radical scavenging capacity (HORAC assay)

The assay was modified according to a report by Ou et al. (2002). Fluorescein (FL) was used as a fluorescent probe. The antioxidant effect was measured by comparing the fluorescence time/intensity area under the curve of the sample to that of a control with no antioxidant. Trolox was used as the standard for calibration.

### Peroxynitrite scavenging capacity (NORAC assay)

Peroxynitrite (ONOO_−_) scavenging values were determined by monitoring the oxidation of DHR-123 based on a protocol by Chung et al. (2001). A stock solution of DHR-123 (5 mM) was prepared in dimethylformamide, purged with nitrogen, and stored at −80°C. A working solution of DHR-123 (final concentration, fc, 5 *μ*mol/L) diluted from the stock solution was placed on ice in the dark before the experiment started. The reaction buffer consisting of 90 mmol/L sodium chloride, 50 mmol/L sodium phosphate (pH 7.4), and 5 mmol/L potassium chloride with 100 *μ*mol/L (fc) diethylenetriaminepentaacetic acid (DTPA) was purged with nitrogen and placed on ice before use. ONOO^−^ scavenging was measured in a fluorescence reader with an excitation wavelength of 485 (20 nm) and emission wavelength of 530 (25 nm). Five minutes after treating with or without SIN-1 (fc 10 *μ*mol/L) or authentic ONOO^−^ (fc 10 *μ*mol/L) in 0.3 N sodium hydroxide, the background and final fluorescent signals were measured. Oxidation of DHR-123 increased by decomposition of SIN-1 gradually, whereas authentic ONOO^−^ rapidly oxidized DHR-123 with its final fluorescent signal being stable over time.

### Superoxide anion scavenging assay (SORAC assay)

The SORAC assay was conducted following the previously described method by Zhang et al. (2009). Hydroethidine (HE) was used to measure O_2_^−^ scavenging capacity. The mixture of xanthine and xanthine oxidase was used to generate O_2_^−^ radicals. Nonfluorescent HE was oxidized by O_2_^−^ to form a species of unknown structure that emits fluorescence signal at 586 nm. Addition of superoxide dismutase (SOD) inhibits the HE oxidation.

### Singlet oxygen scavenging assay (SOAC assay)

The SOAC assay was modified based on the previously described method by Zhao et al. (2003). HE was used as a probe to measure singlet oxygen. The mixture of H_2_O_2_ and MoO_4_^2−^ was used to generate singlet oxygen. About 40 *μ*mol/L solution of HE, 2.635 mmol/L Na_2_MoO_4_, and 13.125 mmol/L H_2_O_2_ working solutions were prepared in N,N-dimethylacetamide (DMA). HE solution (125 *μ*L) was added to a well followed by addition of 25 *μ*L of 2.635 mmol/L Na_2_MoO_4_ and 25 *μ*L of 13.125 mmol/L H_2_O_2_, respectively. Singlet oxygen scavenging was measured in a fluorescence reader with an excitation wavelength of 530 nm and emission wavelength of 620 nm.

### Study design

Twenty-two healthy participants (13 females, nine males) with a mean age of 41 years (range 21–59), and a mean body weight of 77 kg (range 62–92) entered this study. The study was carried out according to the recommendations for clinical trials in humans, declaration of Helsinki. All subjects were in generally good health as confirmed by physical examination and laboratory tests investigating lipid, carbohydrate, and inflammatory profiles (see summary Table in section Results). The study was performed in double-blind, single-dose, crossover, placebo-controlled fashion. Generally accepted contraindications to physical exercise; previously diagnosed type 1 and 2 diabetes; fasting glucose >110; C-reactive proteins >3; liver and kidney impairments; psychiatric disorders, other disorders of acute or chronic nature (gastrointestinal, pulmonary, renal, cardiac, neurological, or psychiatric disorders), known allergies to foods or their ingredients, use of weight reducing preparations or appetite suppressants, *β*-blockers, ACE inhibitors, statins, insulin, NSAID, pain medications, participation in a clinical study within the last 30 days prior to the beginning of this study or during this study as well as intake of vitamins/dietary supplements 2 weeks prior to the start or during the trial were exclusion criteria for participation in this study.

### Study protocol

All 22 participants were separated into the two groups and were matched for age and gender to the best practical and/or possible degree. They received an emotional and general health evaluation questionnaire. At day 0 (requirement), blood was drawn after 12 h of fasting period for performance of laboratory tests and for analysis of glucose as well as extended Vitality test. Standardized breakfast (one bread roll with a glass of water) was served at day 0. Standardized breakfast was also served on day 1 and day 2 along with placebo or SPECTRA™ 100 mg capsule, respectively. Capillary blood was collected for performance of extended Vitality test at the time 0, and also immediately prior to the standardized breakfast on day 0, and prior to the standardized breakfast and treatment on days 1 and 2, as well as after 1, 2, and 3 h after capsule administration. Cardiovascular parameters and blood pressure were recorded using a Dinamap XL (Johnson & Johnson Medical GmbH, Norderstedt, Germany).

### Detection of ROS in human blood

The extended “Vitality” test that we employed in this pilot study was developed by Noxygen Science Transfer & Diagnostics GmbH (Elzach, Germany). The principle of the method is based upon the monitoring of ESR signal of spin probe (CMH, 200 *μ*mol/L) oxidation that has been mixed with freshly drawn blood. During the process, the blood cells stand under the original physiological environment (*t* = 37°C, pO_2_ = 110 mmHg) and remain surrounded by blood plasma that releases biologically available ROS that interacts in intracellular and extracellular space with CMH to form a stable radical CM° (Bassenge et al. 1998; Fink et al. 2000; Mrakic-Sposta et al. 2012). Addition of oxygen-sensitive label (NOX-15.1–5 *μ*mol/L) to the blood sample allows us to monitor oxygen concentrations and cellular as well as mitochondrial oxygen consumption (Bobko et al. 2009; Komarov et al. 2012). Bench-top EPR spectrometer “*nOxyscan*” was used with the following settings: center field: *g* = 2.011, sweep width: 60 G, frequency: 9.76 GHz, power: 20 mW, gain: 1 × 10^3^, modulation amplitude: 1.2 G, sweep time: 5.24 sec, number of scans: 10, number of points: 512, total experimental time: 5 min. Calibration of EPR signal was performed using calibration solution with standard concentration of CM° (10 *μ*mol/L) or oxygen label NOX-063 (5 *μ*mol/L) filled in to 50 *μ*L glass capillary.

### Cellular TNF-*α* response assay

In addition to H_2_O_2_ detection, Noxygen Science Transfer & Diagnostics GmbH has developed and validated an ex vivo cellular inflammatory response assay. Application of this assay provides results describing changes in ROS generation by blood cells after stimulation with external (nonendogenous) TNF-*α*. TNF-*α* had previously been reported to be a key factor of inflammation (Feuerstein et al. 1994). The assay was performed using blood samples from each of the study subjects (20 *μ*L). Samples were not analyzed for changes in TNF-*α* concentration, but rather for changes in downstream effects resulting from exogenous TNF-*α* challenge. Samples were mixed with 20 *μ*L solution of human TNF-*α* (#T6674; Sigma-Aldrich, St. Louis, MO) and spin probe 1-Hydroxy-4-phosphono-oxy-2,2,6,6-tetramethyl-piperidine (PPH, Noxygen GmbH, # NOX-4.1) solved in Krebs Hepes buffer (20 mmol/L, pH 7.4). Final concentrations were 40 ng/mL TNF-*α* and 500 *μ*mol/L PPH, respectively. The mixture filled in a teflon, oxygen permeable capillary tube was placed in the resonator of EPR spectrometer (*nOxyscan*, Noxygen GmbH) equipped with a temperature and gas controller BIO-III (TGC, Noxygen GmbH) for monitoring of EPR signal within 60 min.

The TGC setting was as follows: temperature 37°C, 10 mmHg pressure, and 4% oxygen concentration. EPR settings: center field: 3472 G; sweep width: 60 G; static field: 3458 G; frequency: 9.76 GHz; attenuation: 4.0 dB; microwave power: 20 mW; gain: 1 × 10^3^; modulation frequency: 86.00 kHz; modulation amplitude: 2.2 G; time constant: 40.96 msec; conversion time: 10.24 msec; sweep time: 5.24 sec; number of scans: 10; number of points: 46; experimental time: 60 min. A kinetic curve slope (EPR signal amplitude vs. time) for the 60 min was integrated and expressed as formation of ROS *μ*mol/L per min.

### Bioavailable NO concentration assay

Analysis of circulating NO concentration in human blood, second key signaling molecule of vascular physiology and an in vivo antioxidant, was performed in previous studies (Dikalov and Fink 2005; Pisaneschi et al. 2012).

### Chemicals

The spin probes 1-hydroxy-3-methoxycarbonyl-2.2.5.5-tetramethylpyrrolidine (CMH), 1-hydroxy-4-phosphono-oxy-2.2.6, 6-tetramethylpiperidine (PPH), the metal chelators defferoxamine (DF), and diethyldithiocarbamate (DETC). Krebs–Hepes buffer (KHB), and the oxygen label NOX-15.1 were obtained from Noxygen Science Transfer & Diagnostics, CuZn superoxide dismutase (SOD) was obtained from Sigma–Aldrich (St. Louis, MO). All other chemicals and reagents used were of analytical grade and were purchased from Sigma–Aldrich unless otherwise specified.

### Statistical analysis

All statistical analyses were performed with the SigmaPlot 11.0 (Chicago, IL). *P* value was calculated using one-way ANOVA with Holm-Sidak method, and *P* < 0.05 was considered as statistically significant.

## Results

The biological active phytochemical compounds, vitamins, minerals, and TAC per 100 mg serving size of the SPECTRA™ are summarized in Table1.

**Table 1:** Biological active compounds and TAC for SPECTRA™ per serving size 100 mg

	Units	Result
Phytochemical compound		
Glucosinolates	mg	0.1
Quercetin	mg	10.8
Lycopene	*μ*g	43
Chlorogenic acids	mg	6.7
Vitamin C	mg	1.2
Catechins	mg	10.3
Allicin	*μ*g	10
Alliin	*μ*g	20
Anthocyanins	mg	0.5
Vitamin E	*μ*g	13.4
Beta-carotene	*μ*g	36.4
Folate	*μ*g	1.2
Vitamin K	*μ*g	3.1
Calcium	mg	0.77
Magnesium	mg	0.53
Potassium	mg	4.3
Activity against individual radicals		
Activity against peroxyl radicals	*μ*mol TE	1070
Activity against hydroxyl radicals	*μ*mol TE	1511
Activity against peroxynitrite	*μ*mol TE	110
Activity against superoxide anion	*μ*mol TE	1337
Activity against singlet oxygen	*μ*mol TE	417
Total activity	*μ*mol TE	4445

TAC, total antioxidant capacity.

According to the study protocol, we recruited nine male and 13 female generally healthy participants between the ages of 21 and 59 years and with body weight of 77.4 ± 10.2 kg and BMI of 26.3 ± 2.2 (typical for an industrial country like Germany). Cardiovascular parameters such as heart rate 67 ± 8 bit/min, and blood pressure 123 ± 4 mmHg, did not exceed the values of healthy persons. Mean values of laboratory parameters from all study participants at day “0” are depicted below in the Table2

**Table 2:** Laboratory parameters—hemogram, metabolic-, inflammatory-, and lipid-profile

Hemogram		Inflammatory parameters	
Hemoglobin (g/dL)	14.1 ± 1.3	Leukocytes (tsd/*μ*L)	5.7 ± 1.6
Erythrocytes (mio/*μ*L)	4.7 ± 0.4	Neutrophils absolute (tsd/*μ*L)	3.0 ± 1.0
Hematocrit (%)	40.9 ± 3.6	Neutrophils (%)	52.9 ± 7.5
MCH (pg)	30.1 ± 1.2	Eosinophil absolute (tsd/*μ*L)	0.15 ± 0.10
MCV (fL)	87.1 ± 3.0	Eosinophil (%)	2.5 ± 1.2
MCHC (g/dL)	34.5 ± 0.8	Lymphocytes absolute (tsd/*μ*L)	2.0 ± 0.6
Immature granulocytes (%)	0.3 ± 0.1	Lymphocytes (%)	35.0 ± 6.9
Thrombocytes (tsd/*μ*L)	218.0 ± 52.2	CRP (mg/L)	2.7 ± 2.3

Metabolic-profile		Lipid-profile	
Insulin (mU/L)	10.6 ± 6.5	Triglyceride/neutral fat (mg/dL)	124.8 ± 72.3
Glucose in serum (mg/dL)	90.7 ± 10.2	Cholesterol	217.7 ± 54.7
Glucose/whole blood (mg/dL)	73.2 ± 9.7	HDL cholesterol (mg/dL)	51.7 ± 22.6
Homa-index (mg/dL)	2.3 ± 1.7	VLDL cholesterol (mg/dL)	24.2 ± 14.4
		LDL cholesterol (mg/dL)	141.8 ± 40.1

Detection of ROS was performed with extended Vitality test protocol which allows analysis for:

1. “total”—(extracellular/intracellular) generation of ROS/cellular oxygen consumption;
2. “extracellular”—generation of O_2_^−^/cellular oxygen consumption by addition of SOD (50 U/mL);
3. “extracellular”—generation of H_2_O_2_/cellular oxygen consumption by addition of catalase (50 U/mL);
4. “mitochondrial”—generation of mitochondrial O_2_^−^/mitochondrial oxygen consumption by addition of Antimycin A (10 *μ*mol/L).

**Figure 1:**
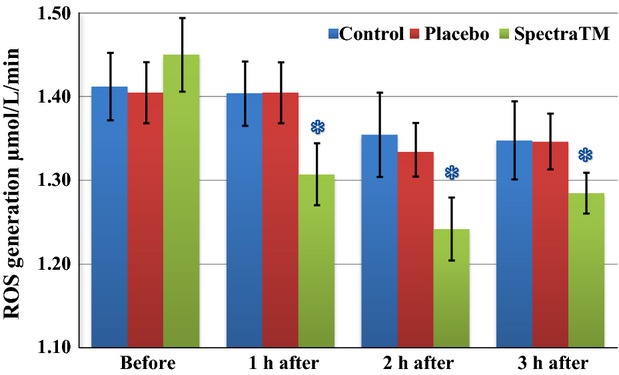
Effect of SPECTRA™ on cellular “total” ROS generation/metabolic activity in human participants. Detection of reactive oxygen species was performed using spin probe CMH (200 *μ*mol/L) and bench-top EPR spectrometer *nOxyscan* in 22 generally healthy, fasted (minimum 12 h) participants. Blue columns (control): prior to and 60, 120, 180 min after consumption of standard breakfast (bread roll with glass of water); red columns (placebo): after consumption of standard breakfast and placebo capsule; and green columns (SPECTRA™): after consumption of standard breakfast and SPECTRA™ capsule. For EPR settings, please refer to material and methods. Data are mean (*n* = 22) ± SEM, *P < 0.05 versus value “before.” CMH, 1-hydroxy-3-methoxycarbonyl-2.2.5.5-tetramethylpyrrolidine; EPR, electron paramagnetic resonance.

**Figure 2:**
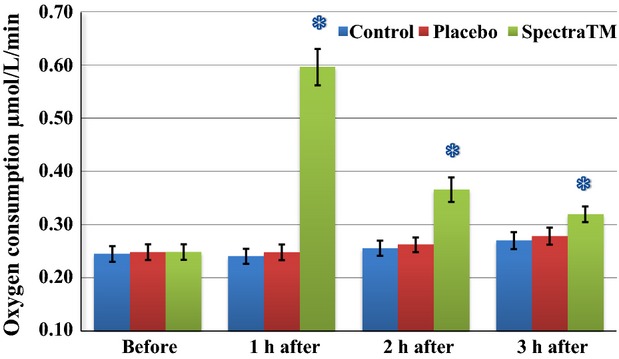
Effect of SPECTRA™ on cellular oxygen consumption of blood cells collected from human volunteers. Oxygen consumption analysis was performed with the same blood samples using spin label NOX-15.1 (5 *μ*mol/L) and bench-top EPR spectrometer *nOxyscan* in 22 generally healthy, fasted (minimum 12 h) participants. Blue columns (control): prior to and 60, 120, 180 min after consumption of standard breakfast (bread roll with glass of water); red columns (placebo): after consumption of standard breakfast and placebo capsule; and Green columns (SPECTRA™): after consumption of standard breakfast and SPECTRA™ capsule. Observation of oxygen concentration changes was possible due to optimization of modulation amplitude 1 G, which makes it possible to follow EPR amplitude of separately appearing CM-radical and oxygen label EPR lines. Data are mean ± SEM (*n* = 22), *P < 0.05 versus value “before.” EPR, electron paramagnetic resonance.

**Figure 3:**
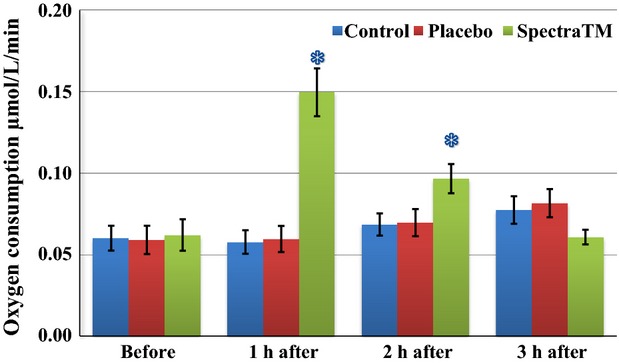
Influence of SPECTRA™ on “mitochondrial” oxygen consumption of blood cells collected from human participants. Mitochondrial oxygen consumption was performed in the same blood samples using spin label NOX-15.1 (5 *μ*mol/L) and bench-top EPR spectrometer *nOxyscan* after addition of Antimycin A (10 *μ*mol/L) prior to and at 60, 120, 180 min after consumption of standard breakfast (bread roll with glass of water). Blue columns (control): prior to and 60, 120, 180 min after consumption of standard breakfast (bread roll with glass of water); red columns (placebo): after consumption of standard breakfast and placebo capsule; and green columns (SPECTRA™): after consumption of standard breakfast and SPECTRA™ capsule. The values of oxygen consumption were calculated as delta value between “total” and “Antimycin A” sample. Data are mean ± SEM, *P* < 0.05 versus value “before.” EPR, electron paramagnetic resonance.

**Figure 4:**
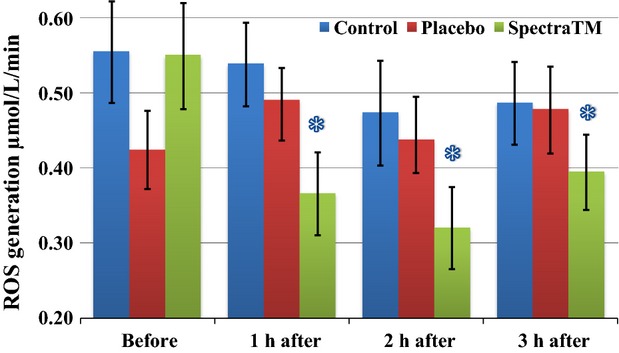
Effect of SPECTRA™ on “mitochondrial” ROS generation in blood cells collected from human volunteers. Detection of mitochondrial ROS generation was performed using spin probe CMH (200 *μ*mol/L) and bench-top EPR spectrometer *nOxyscan* after addition of Antimycin A (10 *μ*mol/L) in the blood samples taken prior to and at 60, 120, 180 min after consumption of standard breakfast (bread roll with glass of water). Blue columns (control): prior to and 60, 120, 180 min after consumption of standard breakfast (bread roll with glass of water); red columns (placebo): after consumption of standard breakfast and placebo capsule; and green columns (SPECTRA™): after consumption of standard breakfast and SPECTRA™ capsule. The values of ROS generation were calculated as delta value between “total” and “Antimycin A” sample. Data are mean ± SEM (*n* = 22), *P < 0.05 versus value “before.” CMH, 1-hydroxy-3-methoxycarbonyl-2.2.5.5-tetramethylpyrrolidine; EPR, elec-tron paramagnetic resonance; ROS, reactive oxygen species.

**Figure 5:**
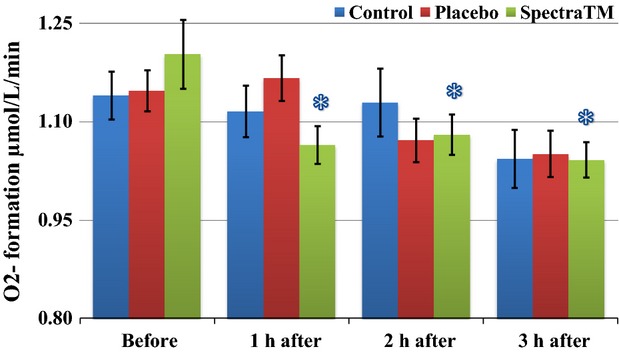
Influence of SPECTRA™ on “extracellular” superoxide (O_2_^−^) formation in blood cells collected from human volunteers. Superoxide formation was analyzed in human blood using EPR spectrometer *nOxyscan*, spin probe CMH (200 *μ*mol/L) after addition of SOD (50 U/mL) in the blood samples taken prior to and at 60, 120, 180 min after consumption of standard breakfast (bread roll with glass of water). Blue columns (control): prior to and 60, 120, 180 min after consumption of standard breakfast (bread roll with glass of water); Red columns (placebo): after consumption of standard breakfast and placebo capsule; and Green columns (SPECTRA™): after consumption of standard breakfast and SPECTRA™ capsule. The values of superoxide generation were calculated as delta value between “total” and “SOD” sample. Data are mean ± SEM (*n* = 22), *P < 0.05 versus value “before.” CMH, 1-hydroxy-3-methoxycarbonyl-2.2.5.5-tetramethylpyrrolidine; EPR, electron paramagnetic resonance.

**Figure 6:**
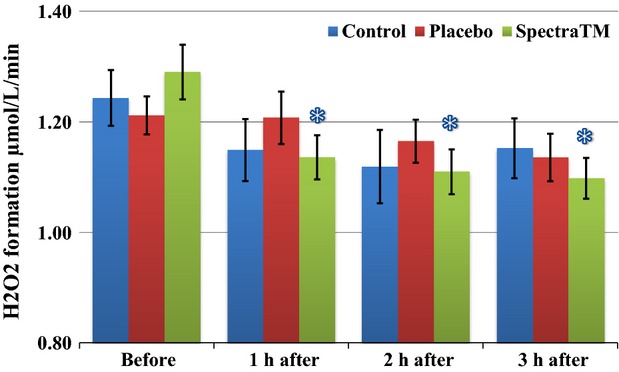
Influence of SPECTRA™ on extracellular H_2_O_2_ formation in blood cells collected from human volunteers. H_2_O_2_ formation was analyzed in human blood using EPR spectrometer *nOxyscan*, spin probe CMH (200 *μ*mol/L) after addition of catalase (50 U/mL) in the blood samples taken prior to and at 60, 120, 180 min after consumption of standard breakfast (bread roll with glass of water). Blue columns (control): prior to and 60, 120, 180 min after consumption of standard breakfast (bread roll with glass of water); red columns (placebo): after consumption of standard breakfast and placebo capsule; and green columns (SPECTRA™): after consumption of standard breakfast and SPECTRA™ capsule. The values of H_2_O_2_ generation were calculated as delta value between “total” and “catalase” sample. Data are mean ± SEM (*n* = 22), *P < 0.05 versus value “before.” CMH, 1-hydroxy-3-methoxycarbonyl-2.2.5.5-tetramethylpyrrolidine; EPR, electron paramagnetic resonance.

As Figure1 immediately below demonstrates, at 60 min after 100 mg single-dose SPECTRA™ administration we observed a significant decrease in total generation of ROS/metabolic activity of blood cells in human volunteers. These effects persisted for another 2 h followed by a significant trend toward baseline 3 h after administration. Under pathological conditions, increased oxidative stress itself can alter oxygen levels. Changes in oxygen levels may subsequently affect mitochondrial oxygen consumption. In order to circumvent this problem, EPR method has been developed for measuring superoxide and oxygen consumption in mitochondrial respiratory complexes using the oxygen labels such as NOX-15.1 (Mariappan et al. 2009). By using a gas controller and nontoxic spin label NOX-15.1, we were able to measure oxygen consumption simultaneously during detection of ROS. The merit of this method is that it allows us to measure superoxide production and oxygen consumption in parallel using the same incubation medium and substrate concentration in each blood sample. Simultaneously to changes in levels of ROS, we observed a significant increase in cellular oxygen consumption (Fig.2) as well as mitochondrial oxygen consumption (Fig.3) after 1 h of SPECTRA™ ingestion. These levels continued to be slightly elevated during the next 2 h. These findings suggest optimization of redox balances and optimization of respiratory activity of mitochondria factors that are important for healthy aging (Dikalova et al. 2010; Nazarewicz et al. 2013). The same pattern as cellular ROS generation was recognized in the mitochondrial generation of ROS after SPECTRA™ administration. It decreased significantly after 1 h and continued to decrease for the next 2 h (Fig.4). Although generation of ROS in the control and placebo group showed nonsignificant tendencies toward depletion of ROS formation, this is consistent with previous observations collected from nonathletically trained participants (B. Fink, unpubl. data). In addition to the decrease in “total” ROS generation and oxygen consumption, the administration of a single dose of SPECTRA™ significantly inhibited generation of extracellular NADPH oxidase-dependent superoxide (O_2_^−^, Fig.5) and peroxidase-dependent hydrogen peroxide (H_2_O_2_, Fig.6). The possibility for such regulatory effects on NADPH-oxidases activity suggests that SPECTRA™ may help support cardiovascular health in healthy aged subjects (Wyche et al. 2004; Dikalov et al. 2012). Compared to the “total” ROS generation, the values of both superoxide and hydrogen peroxide generation in the control as well as in the placebo group showed nonsignificant depletion tendencies over the observation period.

**Figure 7:**
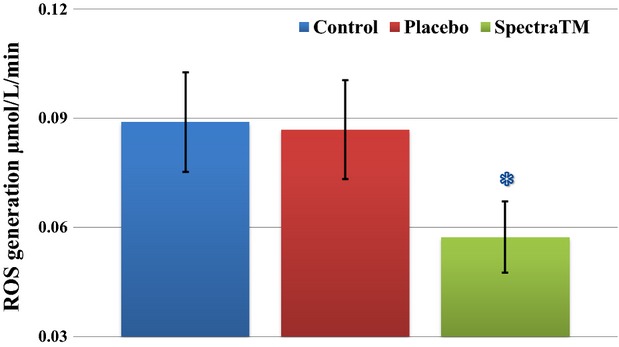
Inhibition of TNF*α*-induced “cellular inflammatory response” after single dose of SPECTRA™ in blood cells collected from human volunteers. This testing measured response of blood cells after chemical insult by stimulation with 40 ng/mL of exogenous human TNF*α*. As expected, this stimulation subsequently induced ROS (H_2_O_2_) formation. Levels of H_2_O_2_ in blood samples from the study subjects were analyzed using EPR spectrometer *nOxyscan*, nonmembrane permeable spin probe PPH (500 *μ*mol/L). Blue column (control): 180 min after consumption of standard breakfast (bread roll with glass of water); red column (placebo): 180 min after consumption of standard breakfast and placebo capsule; and Green column (SPECTRA™): 180 minutes after consumption of standard breakfast and SPECTRA™ capsule. The accumulation of oxidized PP-radical was observed during 1 h incubation at 37C and 40 mmHg oxygen partial pressure. Data are mean ± SEM (*n* = 22), *P* < 0.01 versus placebo. Baseline and posttreatment levels of TNF-*α* were not measured. ROS, reactive oxygen species.

**Figure 8:**
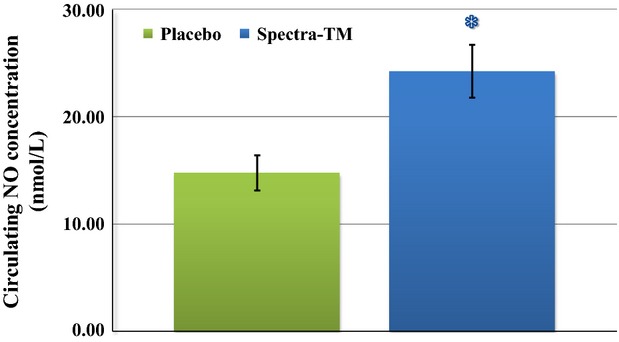
Influence of SPECTRA™ on circulating NO concentration in blood of human volunteers. Bioavailable NO level was analyzed in human blood according to material and methods described by protocol detection of circulating NOHb concentration in blood samples. Green column (placebo): 180 min after consumption of standard breakfast and placebo capsule; and Blue column (SPECTRA™): 180 min after consumption of standard breakfast and SPECTRA™ capsule. Data are mean ± SEM (*n* = 22), *P < 0.01 versus placebo.

In order to provide more robust scientific proof on inhibition of peroxidase activities, which are linked to inflammatory response, we performed analysis of ex vivo changes in cellular ROS (almost hydrogen peroxide, H_2_O_2_) formation after a challenge by stimulation with externally introduced TNF*α*. TNF*α* is recognized as one of the key mediators of inflammation that is directly linked to ROS generation and apoptosis. We demonstrated significant inhibition of cellular response after administration of SPECTRA™ (Fig.7). Another example of the multifaceted effect of SPECTRA™, especially in terms of potential for support of cardiovascular health, is normalization of “nitrosative stress,” which may be evaluated based on analysis of bioavailable circulating NO concentration in vivo (Pisaneschi et al. 2012). Detection of circulating NO concentration in whole blood of participants after administration of SPECTRA™ showed significant increase in the level of NO (Fig.8).

## Discussion

In this pilot study, we delivered evidence expanding on the introduction of the free radical theory of aging proposed by Harman (1956). Thereafter, in 1969, the discovery of the enzyme superoxide dismutase (SOD), provided further convincing evidence suggesting the importance of healthy levels of free radicals in living systems (McCord and Fridovich 1969), and the possible use of nutritional supplements to maintain “optimal health” by modulating the extent of “oxidative and nitrosative stress.” The observed multifaceted biological effects of SPECTRA™ on “oxidative and nitrosative stress” may be directly attributable to the supplement's biologically active compounds as well as substrates required for healthy function of enzymes involved in redox regulation reported in Table1.

The previously reported cardiosupportive action of quercetin, one of the major active compounds in SPECTRA™, was described as a compound attenuating oxidative stress by depletion of serum and tissue MDA (malondialdehyde) formation and moderate incrementation of antioxidant reserves (Annapurna et al. 2009). Such cardiosupportive effects may be caused by inhibition of mitochondrial ROS generation, which have been demonstrated in this clinical study (Fig.4). Possible explanation of that mechanism was proposed by Chen et al. (2013) wherein it was reported that inhibition of doxorubicin-dependent cardiomyocyte oxidative damage was caused by uncoupling of mitochondria. Another cardiosupportive mechanism of quercetin has been suggested in recent studies that reported the inhibitory effects of quercetin on inducible NO synthase over TNF-*α* and on inflammatory gene expression (Wadsworth and Koop 2001; Wadsworth et al. 2001; Boesch-Saadatmandi et al. 2011). It has been shown that these effects as well as predisposition of inflammatory cascade components begin with phenotypic differences in redox-enzymes such as NADPH oxidase (Wyche et al. 2004), GSH reductase (Bailey et al. 2014), catalase (Suvorava et al. 2005), heme oxygenase (Seo et al. 2013) and play an important role in inflammatory responses. In this study, we observed the modulatory effect of SPECTRA™ on increases in ROS generation brought about due to challenge with exogenous TNF-*α* (Fig.7) as well as on preservation of bioavailable NO by reduction in cellular and mitochondrial ROS formation (Fig.8).

Tea polyphenols known as catechins are present in two SPECTRA™ components—green tea extract and apple extract. Previous studies (Yamamoto et al. 2004; Manach et al. 2005) reported that catechins may increase the antioxidant capacity of human plasma, which in turn could support cardiovascular health, improvement of processes associated with lipoprotein oxidation, blood aggregation, and changes in lipid profiles. Other studies (Imai and Nakachi 1995; Sesso et al. 1999; Nakachi et al. 2000; Sasazuki et al. 2000; Sano et al. 2004; Kuriyama et al. 2006; Kuriyama 2008) also suggested that tea polyphenols consumption may promote cardiovascular health due to activation of CuZn-SOD activity and by increasing enzyme expression a parameter also observed by SOD-dependent extracellular O_2_^−^ generation (Fig.5) in this current study.

Earlier science has suggested that dietary chlorogenic acids (CGA)—the major group of coffee polyphenols—may reduce the oxidative stress and improve nitric oxide bioavailability by inhibiting excessive production of reactive oxygen species in the vasculature, and lead to the attenuation of endothelial dysfunction (Ohga et al. 2009; Yan et al. 2013). Others reported that the initial CGA metabolite, caffeic acid, significantly increased superoxide dismutase, catalase, and glutathione peroxidase activity and lowered plasma glucose concentration (Rustan et al. 1997; Jung et al. 2006). CGA has also been examined in human studies for possible effects upon blood pressure and vasoreactivity effects (Watanabe et al. 2006).

Observation of allicin showed spontaneous inhibition and TNF-*α* induced secretion of proinflammatory cytokines and chemokines from intestinal epithelial cells (Lang et al. 2004). Allicin can permeate epithelial and red blood cells membranes of phospholipids bilayers, carry out its activity intracellularly, and interact with SH groups (Miron et al. 2000).

Biologically active compounds as well as microelements, vitamins, and enzymes in natural supplement SPECTRA™ may participate and support the regulation of degree from “oxidative and nitrosative stress.” Previous clinical studies have reported that administration of vitamin C (Bassenge et al. 1998) or vitamin E (Mah et al. 2013) in low dosages were able to inhibit/restore endothelial dysfunction, a consequence of excessive “oxidative and nitrosative stress.” Therefore, it may be possible that components of SPECTRA™ may contribute in unfolding activity of biologically active enzymes.

Initially, the Total ORAC_FN_ assay was used to determine SPECTRA™'s ability to modulate the in vitro antioxidant scavenging capacity of five major free radicals (peroxyl, superoxide anion, hydroxyl, singlet oxygen, and peroxynitrite) that are naturally produced in the body. This product has been standardized to deliver a total minimum ORAC_FN_ of 40,000 *μ*mol TE/g. In order to confirm that SPECTRA™ could exert any activity in vivo, we employed the extended “Vitality” test, which allows us to measure the influence of SPECTRA™ on all four components of the “healthy aging hypothesis.” The strength of the signals generated by cyclic hydroxylamines and the ability to use the technology to study changes that occur at the level of cellular components such as mitochondria, vessels, cells, and human blood (Fink et al. 2000; Mrakic-Sposta et al. 2012) allow us to follow quantifiable biological effects in healthy human subjects. CMH was adopted due to the fact that it is a molecule capable of diffusion in cell compartments, including mitochondria (Dikalov et al. 2011). Indeed, due to its particular physical-chemical properties, the CMH probe is able to cross biological membranes, thereby detecting ROS both in plasma and intracellular compartments. In this way, EPR measurements enable us to make relative quantitative determinations of ROS production rates in human blood samples. Additionally, owing to its high efficiency in radical detection, CMH probe can be used at very low concentrations (0.2 mmol/L) compared to spin traps (10–50 mmol/L), an attribute that minimizes side-effects of the probes on cell physiology. Moreover, CMH rapidly reacts and allows radical detection via a single chemical reaction, while other probes require at least two reactions that may cause artifacts by interaction of two byproducts (Zielonka et al. 2005).

**Figure 9:**
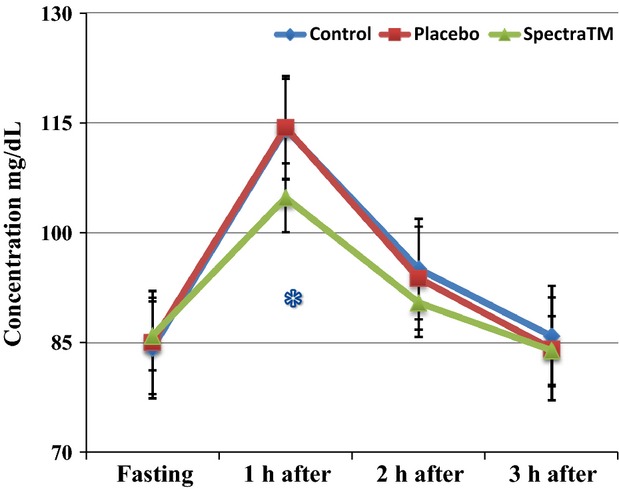
Changes in blood glucose concentration after supplementation of standard breakfast with or without placebo/SPECTRA™. Data are mean ± SEM (*n* = 22), *P < 0.025 versus placebo.

In addition to the above-reported biological effects on oxidative and nitrosative parameters, we observed an additional effect of lowering of glucose concentrations in the participants' blood (Fig.9) that may have been associated with increases of mitochondrial oxygen consumption as well as metabolic activity of cells. Such possible effect of SPECTRA™ is worthy of further investigation.

## Conclusions

For the first time, we were able to measure the biological effects of a natural dietary supplement on changes of “oxidative and nitrosative stress markers” and cellular metabolic activity through the use of the extended “Vitality Test.” Unique activity of SPECTRA™ suggests potential for the use of the supplement in modulation of oxidative stress, NO bioavailability, inflammatory response, blood glucose levels, and ultimately supporting “optimal health.”
